# Effects of Intermittent Hypoxia on Cytokine Expression Involved in Insulin Resistance

**DOI:** 10.3390/ijms222312898

**Published:** 2021-11-29

**Authors:** Tomoko Uchiyama, Hiroyo Ota, Chiho Ohbayashi, Shin Takasawa

**Affiliations:** 1 Department of Biochemistry, Nara Medical University, Kashihara 634-8521, Japan; shintksw@naramed-u.ac.jp; 2 Department of Diagnostic Pathology, Nara Medical University, Kashihara 634-8522, Japan; ohbayashi@naramed-u.ac.jp; 3 Department of Respiratory Medicine, Nara Medical University, Kashihara 634-8522, Japan; hiroyon@naramed-u.ac.jp

**Keywords:** intermittent hypoxia, sleep apnea syndrome, insulin resistance, hepatokines, adipokines, myokines

## Abstract

Sleep apnea syndrome (SAS) is a prevalent disorder characterized by recurrent apnea or hypoxia episodes leading to intermittent hypoxia (IH) and arousals during sleep. Currently, the relationship between SAS and metabolic diseases is being actively analyzed, and SAS is considered to be an independent risk factor for the development and progression of insulin resistance/type 2 diabetes (T2DM). Accumulating evidence suggests that the short cycles of decreased oxygen saturation and rapid reoxygenation, a typical feature of SAS, contribute to the development of glucose intolerance and insulin resistance. In addition to IH, several pathological conditions may also contribute to insulin resistance, including sympathetic nervous system hyperactivity, oxidative stress, vascular endothelial dysfunction, and the activation of inflammatory cytokines. However, the detailed mechanism by which IH induces insulin resistance in SAS patients has not been fully revealed. We have previously reported that IH stress may exacerbate insulin resistance/T2DM, especially in hepatocytes, adipocytes, and skeletal muscle cells, by causing abnormal cytokine expression/secretion from each cell. Adipose tissues, skeletal muscle, and the liver are the main endocrine organs producing hepatokines, adipokines, and myokines, respectively. In this review, we focus on the effect of IH on hepatokine, adipokine, and myokine expression.

## 1. Introduction

Sleep apnea syndrome (SAS) is a common disorder that causes repeated apnea and hypopnea due to the obstruction of the upper airway during sleep, and it occurs in about 13–33% of men and 5–19% of women [1,2]. Repeated nocturnal obstruction of the upper airways, a characteristic of SAS, lead to the following physiological effects: fluctuating intrathoracic pressure, sleep fragmentation, hypercapnia, and intermittent hypoxia (IH) [3]. The involvement of these pathologies can lead to chronic diseases of multiple organs. A number of conditions may occur including atherosclerosis, cardiovascular disease, cerebrovascular disease, immunodeficiency, and metabolic abnormalities (pancreatic β cell dysfunction, insulin resistance, and increased free-fatty acid [FFA]) may occur [4,5,6,7,8,9,10,11].

In this review, we focus especially on IH because SAS patients are exposed to IH to repeat anoxia periodically by night. IH induces ischemia-reperfusion (I/R) injury due to a fluctuating situation where hypoxia and reoxygenation occur repeatedly [12]. IH is a key manifestation of SAS and is thought to initiate a cascade of pathological conditions characterized by sympathetic activation, mitochondrial dysfunction, inflammation, oxidative stress, endothelial dysfunction, and metabolic disorders [3,11,13]. SAS patients may display one or more symptoms including fragmented sleep, snoring, excessive daytime sleepiness, fatigue, high blood pressure, depression, and loss of concentration.

SAS and type 2 diabetes (T2DM) are known to be closely associated with each other. About 15–30% of SAS patients have T2DM, and the majority of T2DM patients also suffer from obstructive sleep apnea [14,15]. Perhaps the most important risk factor for SAS is obesity; however, only about 60–70% of sleep apnea patients are obese [3,16]. The association between SAS and insulin resistance is independent of other factors such as obesity, age, and sex [14,17,18,19,20,21], and IH itself is considered to be a risk factor for insulin resistance/T2DM [5,7,16,19,22,23,24,25]. In recent years, Michalek-Zrabkowska et al. investigated the relationship between the severity of SAS without comorbid T2DM and insulin resistance. They concluded that even in the absence of T2DM comorbidity, there is the suggestion of an association with insulin resistance in patients with moderate to severe SAS. Similar findings have been reported in other studies [17,26].

Chronic IH with reoxygenation induces a variety of pathogenesis similar to I/R injury and is thought to play a major role in the diseases caused by SAS [27]. Additionally, various mechanisms leading to glucose intolerance via IH have been reported: sympathetic activation due to hypoxic stress, and pancreatic β cell dysfunction because of oxidative stress, and the involvement of inflammatory cytokines [22,24]. Other organs including the liver [5,19], adipose tissue [7,19,28,29,30], skeletal muscle [31], the central nervous system [32], and the gastrointestinal tract [25,32] are considered to be associated with glucose intolerance/insulin resistance due to IH. A direct effect of IH is an oxidative imbalance in the production of reactive oxygen species (ROS) and the activation of the inflammatory cascade by increased proinflammatory and anti-inflammatory cytokines; for example, tumor necrosis factor-α (TNF-α), interleukin (IL)-1, and IL-6 are well known. However, many details remain to be clarified about the expression of cytokines under the influence of IH, including the organs of origin and their effects [33].

The relationship between IH and cytokine secretion has been reported not only in relation to insulin resistance and glucose intolerance, which is reviewed in this article, but also in relation to abnormal cytokine secretion associated with cardiovascular and renal diseases, nasal mucosal injury, and inflammation of the upper respiratory tract [34,35,36,37]. Since we previously investigated the way in which IH induces impaired insulin secretion/insulin resistance in in vitro experimental systems using pancreatic β cells, neuronal cells, hepatocytes, adipocytes, skeletal muscle cells, and cardiomyocytes [5,7,19,25,32,38], in this review, we focus on how IH induces insulin resistance.

Specifically, we describe changes in cytokines secreted by organs affected by IH, particularly the liver (hepatokines), adipose tissue (adipokines), and skeletal muscle (myokines), and their relationship to insulin resistance.

## 2. Intermittent Hypoxia and Cytokines

### 2.1. Intermittent Hypoxia and Hepatokines

It has long been known that there is a close relationship between glucose intolerance and liver disease. In the insulin-resistant state, lipolysis in adipose tissue is enhanced, leading to increased FFA in the blood and increased FFA influx into the liver. The pancreatic β cells compensatively increase insulin synthesis and secretion, resulting in hyperinsulinemia and increased synthesis of new fatty acids in the liver, leading to a nonalcoholic fatty liver disease (NAFLD). The excessive accumulation of triglycerides in the liver induces oxidative stress and inflammation, leading to the development of nonalcoholic steatohepatitis (NASH). In addition, abnormalities in hepatic lipid metabolism caused by insulin resistance lead to increased secretion of atherosclerosis-inducing lipoprotein abnormalities, inflammatory cytokines, and blood clot promoting factor, which promote atherosclerosis and create high-risk conditions for cardiovascular diseases [8,39].

Obstructive sleep apnea syndrome (OSAS) has recently been linked to NAFLD, the most common chronic liver disease in the world, which is found in about 25% of the general adult population and up to 75% of obese people [3,40,41]. According to some studies, chronic IH in patients with OSAS may itself cause liver injury, inflammation, and fibrosis, promoting the development of NAFLD and progression from lipidosis to steatohepatitis, cirrhosis, and hepatocellular carcinoma. In patients with NAFLD, IH may cause liver disease indirectly by promoting inflammation and insulin resistance and directly by promoting the production of inflammatory cytokines and metabolic abnormalities in hepatocytes [3,42,43,44,45,46,47].

**Figure 1:**
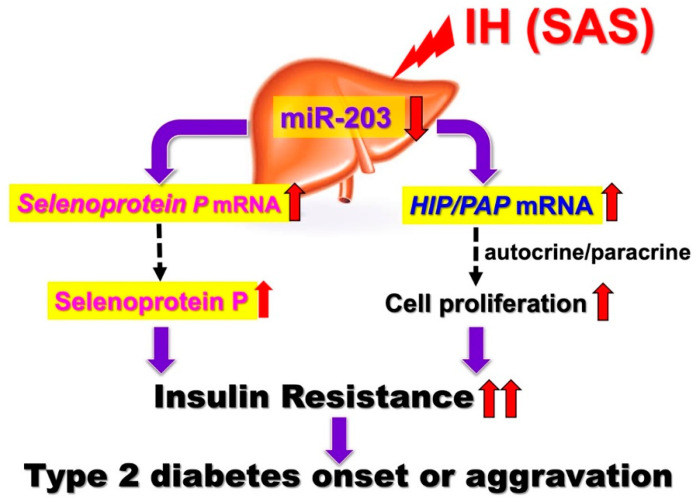
IH-induced upregulation of SeP and HIP/PAP via downregulation of miR-203. IH stress upregulates the levels of *SeP* in human hepatocytes to accelerate insulin resistance and upregulates the levels of HIP/PAP mRNAs to cause such hepatocytes to proliferate, via the miR-203 mediated mechanism. [5]. In SAS patients, it is suggested that the upregulation of SeP plays a role in worsening insulin resistance. In addition, overexpression of HIP/PAP could cause such hepatocytes to proliferate in SAS patients, leading to decreased insulin sensitivity. Red up and down arrows mean increment and decrease, respectively.

A few studies investigated the relationship between IH and cytokines released from the liver (Figure 1). Briancon-Marjollet et al. investigated the respective effects of obesity and IH on inflammatory and cardiometabolic state in rats by exposing lean and obese rats to normoxic conditions or chronic IH and evaluating their serum leptin (LEP), adiponectin (ADIPOQ), hepatic cytokines, nuclear factor-κB (NF-κB) activity, and cardiac endothelin-1 levels. The results showed that the levels of IL-6 and TNF-α in the liver were elevated in lean rats exposed to IH [21]. Mesarwi et al. published the following research findings on the association of IH with the progression of NAFLD. NAFLD with steatohepatitis was induced in mice with the hepatocyte-specific deletion of hypoxia-inducible factor (Hif)1α and in wild-type control mice. The mice were then divided into those with IH and a control group. The HIF1α-deficient mice showed less weight gain and improved fasting blood glucose and insulin resistance. The collagen in the liver increased in the IH group and decreased in the HIF1α-deficient mice. Based on these results, they concluded that HIF-1 signaling worsens the metabolic profile, accelerates the progression of NAFLD, and exacerbates liver fibrosis [48]. The activation of HIF-1 under IH is thought to occur as a result of severe hypoxemia or the excessive production of ROS during the hypoxia-reoxygenation associated with apnea-hypopnea [49]. Zhou et al. investigated the association between NAFLD and SAS in children, using a mouse model divided into Western diet (WD) and control groups and an IH and room air group for 6–12 weeks. The results showed that the WD and IH groups resembled the histological characteristics of pediatric NASH. In addition, the levels of IL-1β, IL-6, and IL-18 were elevated in the WD and IH groups, suggesting that they promote the inflammatory response in the liver [46].

Recently, as with adipose tissue, the pancreas, and skeletal muscle, the liver has been recognized as an endocrine organ that secretes a protein known as hepatokine, a liver-derived factor that can signal and transmit to distant tissues and affect glucose metabolism. The liver senses nutrient excess and deficiency and secretes hepatokine and liver-derived factors to regulate nutrient availability to peripheral tissues and the central nervous system [8,50]. The relationship between hepatokine secreted by the liver and insulin resistance has been reported, and we examined the effects of IH on hepatokine expression and hepatocyte proliferation in vitro. Selenoprotein P (SeP encoded by the *SEPP1* or *SELENOP* gene) is a liver-derived selenium carrier protein that is closely related to glucose metabolism. SeP blood levels are known to be high in patients with NAFLD, obesity, and diabetes, and elevated SeP levels are thought to be involved in insulin resistance [46,51,52]. Our in vitro study revealed that the gene expression of SeP and hepatocarcinoma-intestine-pancreas/pancreatitis-associated protein (HIP/PAP) were increased via the downregulation of the miR-203 level in IH-treated hepatocytes. The correlation between IH and epigenetic regulation is discussed below. We also studied the *angiopoietin-like protein* (*ANGPTL*) 6, 8, *sex hormone-binding globulin* (*SHBG*), *fibroblast growth factor* (*FGF21*), *leukocyte cell-derived chemotaxin* (*LECT*) 2, and *alpha-2-HS-glycoprotein* (*AHSG*) mRNA levels in IH-treated hepatocytes, and we observed no significant change common to all the analyzed hepatocytes [5]. There are a few studies on IH and hepatokine secretion aside from ours, especially in in vitro experimental systems.

The regenerating gene (Reg) was identified in regenerating islets [53,54,55] and a Reg gene product—Reg protein—acts as a growth factor and promotes cell proliferation and regeneration [53,56,57]. In humans, five functional Reg family genes (REG Iα, REG Iβ, REG III, HIP/PAP, and REG IV) have been isolated. For several cells, it has been suggested that Reg family proteins are involved in cellular proliferation [53,55]. Ota et al. reported that IH stress stimulates pancreatic β cell proliferation via the up-regulation of Reg family mRNAs and may cause hyperinsulinemia, which makes patients more obese [24]. However, the direct effects of IH on hepatocyte proliferation and the IH-induced changes in Reg family gene expression in hepatocytes remain unknown. We analyzed the changes of REG family gene expression in human hepatocytes by IH. The mRNA levels of HIP/PAP were significantly increased in IH-treated human hepatocytes [5]. Meanwhile, the mRNA levels of the other REG family members (REG Iα, REG Iβ, REG III, and REG IV) were not increased. Human hepatocytes (HepG2, JHH5, and JHH7 cells) were exposed to normoxia or IH for 24 h. HIP/PAP is originally found as a gene overexpressed in liver carcinoma [58] and the pancreas during the acute phase of pancreatitis [59]. Recent HIP/PAP transgenic/knockout mouse experiments [60,61,62] revealed that it is an autocrine/paracrine mitogenic factor, accelerating liver regeneration. HIP/PAP promotes cell proliferation in hepatocytes by protecting them from apoptosis and acting as a mitogenic factor [60,62]. After the IH treatment of HepG2 cells, cell viability was determined by WST-8 assay. HepG2 cell proliferation was significantly increased by IH. Furthermore, the RNA interference of HIP/PAP inhibited HepG2 cell proliferation measured by WST-8 assay, whereas the interference of other REG family genes such as REG Iα did not. These results indicate that HIP/PAP, upregulated by IH, works as an autocrine/paracrine growth factor in hepatocyte proliferation. In addition, the IH-induced upregulation of SeP and HIP/PAP was revealed to be mediated via the downregulation of miR-203 in hepatocytes [5]. In SAS patients, it is suggested that the upregulation of SeP plays a role in worsening insulin resistance (Figure 1).

In addition, the overexpression of HIP/PAP could cause such hepatocytes to proliferate in SAS patients, leading to decreased insulin sensitivity. Moreover, Takeda et al. recently reported that the expression of renin in juxtaglomerular cells was significantly increased in response to IH stimulation via the downregulation of miR-203 [63]. The most common complications in SAS patients are hypertension and diabetes, and IH, caused by SAS, reduced miR-203 in hepatocytes [5] and juxtaglomerular cells [63], resulting in increased Sep in hepatocytes, a diabetogenic hepatokine, and renin in juxtaglomerular cells, which induces hypertension, simultaneously.

### 2.2. Intermittent Hypoxia and Adipokines

Adipose tissue is a complex tissue composed of preadipocytes, adipocytes, and interstitial vascular cells and is one of the major organs that contribute to worsening insulin resistance through inflammation and subsequent impaired function. It has long been known that the inflammation and dysfunction of adipose tissue play a pivotal role in the pathogenesis of diabetes. Adipocytes not only convert excess energy into triglycerides and store them but also express and secrete adipokines as endocrine organs to control and regulate the metabolism of the whole body. Numerous studies have been undertaken on the mechanisms of adipose tissue dysfunction in obese patients. In obesity, adipocytes hyperplasia and hypertrophy may occur [16,30,64]; however, obesity not only leads to increased fat storage but also to the abnormal function of adipose tissue as an endocrine organ. Adipocyte hypertrophy leads to functional abnormalities and inflammation, resulting in insulin resistance and immune cell infiltration, localized hypoxia, and fibrosis. Following the infiltration of CD8-positive cytotoxic T cells and the phenotypic change to M1 macrophages, inflammatory adipokines including TNF-α, IL-6, and RETN are produced, leading to insulin resistance [16,30,65,66]. Additionally, hypoxia is thought to be an important factor in adipose tissue dysfunction in obesity. Continued adipocyte hypertrophy leads to local hypoxia and the activation of the hypoxia-inducible transcription factors, in particular HIF-1, which is regarded as the key molecule for signaling the cellular response to low oxygen levels, thus contributing to dysfunctions and metabolic disorders in adipocytes [67].

Previous studies conducted under IH conditions have reported a decrease in mass and cell shrinkage in adipocytes [30,68,69]. Although the adipocyte morphology under obese and IH-exposed conditions may differ, some of the dysfunction mechanisms are common to both [68,70]. The mechanism of inflammatory cell infiltration and other mechanisms (such as sympathetic activation and the exacerbation of oxidative stress) are assumed to be common pathways. Due to sympathetic activation, IH cause an increase in blood catecholamines [71], which in turn causes lipolysis and produces FFA, leading to insulin resistance in skeletal muscle and liver cells [72,73]. The chronic elevation of FFA levels has an adverse effect on glucose homeostasis, called lipotoxicity [74]. In addition, in the IH environment, HIF activation by adipose tissue hypoxia has been reported to worsen insulin sensitivity [68], and HIF-1α protein expression in the blood is reportedly significantly elevated in SAS patients [75,76]. Some reports suggest that HIF-1 is activated in sustained hypoxia (SH), but not in IH, and that inflammatory pathways are selectively activated [77]. In the IH environment, both HIF-1 and NF-κB are activated, leading to changes in gene expression. Since HIF-1 is a direct target, such as being transcriptionally regulated by NF-κB, and TNF-α is also regulated by NF-κB, it is possible that crosstalk between HIF-1 and NF-κB is associated with cytokine abnormalities under IH, but the detailed mechanism is not known [77,78]. As described above, there are several possible mechanisms by which IH induces insulin resistance and not all of them have been elucidated. We conducted research focusing on adipokines.

Adipokines are multiple hormones, cytokines, chemokines, and other proteins secreted by adipocytes in white adipose tissue [30]. Some adipokines are specific to adipocytes, and some are not. More than 50 types of adipokines have been identified to date, including TNF-α and IL-6, which are known to be factors that increase the risk of diabetes and cardiovascular disease [28,29,30]. The most common adipokines involved in insulin resistance include leptin (LEP), which is involved in appetite control and hypersensitivity to insulin, ADIPOQ, which promotes glucose uptake in skeletal muscle and liver, resistin (RETN), which exacerbates insulin resistance, and C-C motif chemokine ligand 2 (CCL2), which is involved in immunoregulatory and inflammatory processes and is a critical factor for monocyte infiltration [28,30,67,79,80], ANGPTL, which regulates glucose homeostasis, lipid metabolism, and insulin sensitivity [49,81], plasminogen activator inhibitor-1 (PAI-1), and vascular endothelial growth factor (VEGF), which is linked to the inflammatory response [67].

The detailed mechanism of association adipokines with SAS remains unclear. Recently, it was reported that FFA and inflammatory mediators such as TNF-α in serum are elevated in SAS patients and mice in an experimental IH environment, and several in vivo studies reported that IH causes adipose tissue inflammation and insulin resistance [68], suggesting that IH may enhance inflammation and dysfunction in adipose tissue [13,16].

**Figure 2:**
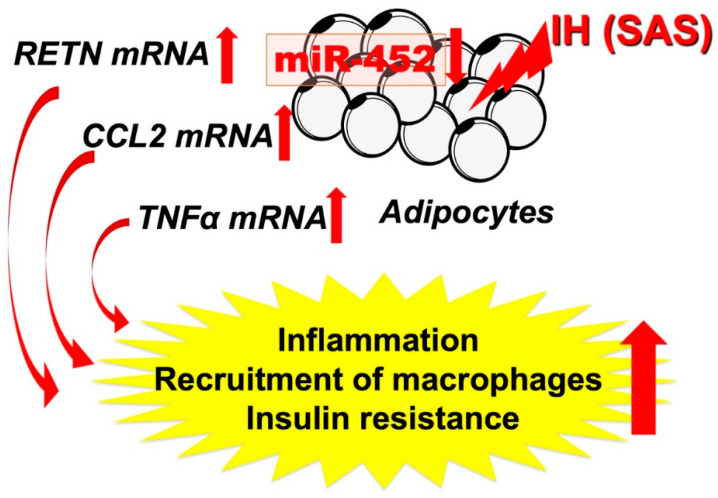
IH-induced increases in RETN, TNFα, and CCL2 via downregulates the miR-452 in adipocytes. It is suggested that, in SAS patients, the upregulation of *RETN*, *TNFα*, and *CCL2* [7] may induce a proinflammatory phenotype of the adipose tissue, leading to the development of insulin resistance and reduced insulin sensitivity, and miR-452 could play crucial roles in the regulation of these gene expressions. Red up and down arrows mean increment and decrease, respectively.

Recently, we investigated the expression and regulation mechanism of adipokines by employing IH exposure. Therefore, to clarify the mechanism by which IH induces inflammation in adipose tissue and exacerbates insulin resistance, we examined the effects of IH exposure on TNF-α, IL-6, etc. A total of seven molecules were measured: six adipokines (ADIPOQ, RETN, LEP, CCL2, TNF-α, and IL-6), which are molecules involved in insulin resistance or whose expression is expected to be altered under IH conditions, and uncoupling protein-1 (UCP-1), which is secreted by brown adipocytes and promotes heat production. The mRNA levels of RETN, TNF-α, and CCL2 were shown to be increased in mouse 3T3-L1 and human SW872 adipocytes by IH exposure. These results suggest that IH in SAS patients is associated with adipocyte inflammation and the worsening of insulin resistance. We further investigated the mechanism by which IH increases the mRNA levels of adipokines such as RETN, TNFα, and CCL2, and found a possible post-transcriptional regulation by miR-452 [7,19]. As shown in Figure 2, the abnormal secretion of TNF-α, IL-6, and CCL2, etc., is suggested in adipose tissue under IH conditions, including in our study. Ge et al. have shown that macrophages accumulate in adipose tissue when lean mice are exposed to IH and in obese mice [82]. CCL2 may induce monocytes into adipose tissue and induce inflammation in an IH environment. Although not all SAS patients are obese, exposure to IH may exacerbate insulin resistance by a mechanism similar to that found with obesity under conditions involving adipokines. Recently, Akasaka et al. reported that advanced glycation endproduct, high-mobility group box 1, and lipopolysaccharide in pregnant women upregulate the expression of IL-6 and CCL2 in adipocytes, leading to systemic inflammation such as preeclampsia/hypertensive disorders of pregnancy [19]. As an association between sleep-disordered breathing, gestational hypertension, and preeclampsia has been demonstrated [83,84,85], CCL2 expressed and secreted from patient adipocytes may link between SAS and hypertensive disorders of pregnancy/gestational diabetes.

### 2.3. Intermittent Hypoxia and Myokines

Myokines, a comprehensive term for cytokines secreted by skeletal muscle, are released from skeletal muscle during muscle contraction [8,86]. The roles of myokines are diverse and include metabolic regulation, anti-inflammatory effects, and the regulation of skeletal muscle mass during injury regeneration [8,87]. Myokines include proteins, microRNA (miRNA)s, and exosomes, and hundreds of them have been found, but only a few of their biological functions have been elucidated. These myokines exhibit autocrine/paracrine and endocrine effects and may mediate the beneficial effects of exercise on other major organs involved in the regulation of energy homeostasis [87,88]. Adipose tissue appears to be an important target for myokines, which regulate energy flux and fuel supply during muscle contraction [8,87]. IL-6 was the first myokine to be discovered, and the level of circulating IL-6 secreted by skeletal muscle is markedly elevated during exercise and muscle contraction. Some clinical studies have shown that IL-6 is needed to reduce visceral adipose tissue during exercise [89], which in turn may lead to improved insulin resistance. Park T.J. et al. found that myonectin (MN) inhibits adipogenesis in 3T3-L1 preadipocytes by downregulating the expression of adipogenic transcription factors such as CCAAT/enhancer binding protein (C/EBP) α, β, and peroxisome proliferator-activated receptor (PPAR) γ. Furthermore, they showed that MN has an inhibitory effect on adipogenesis through the regulation of the p38 mitogen-activated protein kinase (MAPK) pathway and C/EBP homologous protein (CHOP). These results suggest that MN may be a new therapeutic target for obesity prevention [90] and required for metabolic homeostasis [91]. In addition, there are various other myokines that are secreted by skeletal muscle and influence pancreatic, hepatic, and adipose tissue and that are thought to affect glucose tolerance through various mechanisms. Many myokines act not only on other organs but also on themselves (autocrine), leading to the hypertrophy of skeletal muscle and increased insulin sensitivity (increased glucose uptake). Myokines such as myostatin, on the other hand, act on the self (autocrine), causing a decrease in skeletal muscle mass, worsening insulin resistance and promoting fat deposition in the liver [8]. Myokines are involved in the anti-inflammatory effects of physical activity and counteract the metabolic abnormalities of insulin resistance and diabetes [92,93]. Therefore, abnormalities in myokine secretion and function may have a direct influence on the worsening of insulin resistance. However, the mechanism by which myokines affect insulin resistance has not been fully elucidated [87,92,93,94].

**Figure 3:**
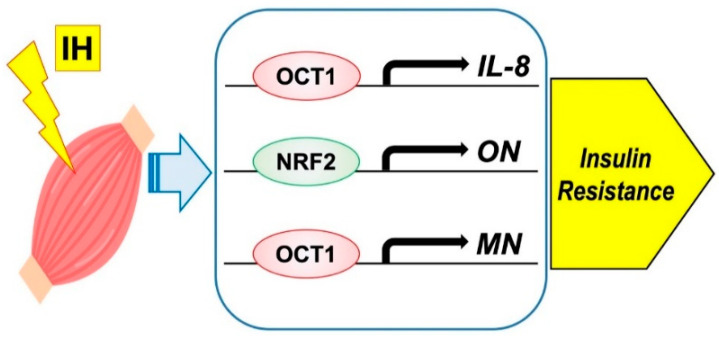
Possible mechanism on IH-induced insulin resistance via myokine expression in muscle cells. IH significantly increased the mRNA levels of *IL-8*, *ON*, and *MN*. The promoters contain consensus transcription factor binding sequences Figure 1 in *IL-8* and *MN*, and for NRF2 in *ON*, respectively.

Moreover, few studies have examined the direct effect of IH on myokine secretion. Otaka et al. examined the role of MN in myocardial ischemic injury by using knockout mice subjected to I/R conditions and found that MN increased cardiac dysfunction, apoptosis, and inflammatory gene expression more than with wild-type mice. On the other hand, transgenic mice overexpressing MN showed less myocardial damage after I/R, indicating that MN functions as an endurance exercise-induced myokine and ameliorates acute myocardial ischemic injury by suppressing apoptosis and inflammation in the heart [95]. I/R is characterized by a limited blood supply to the organs and subsequent tissue damage as a result of recovery. Many studies have shown that inflammation associated with I/R injury can exacerbate myocardial damage [96], which may cause a condition experimentally similar to IH. On the basis of these studies [97], we suspected that IH had a direct effect on myokine secretion and conducted the following experiments. We investigated the changes in myokine levels and their regulation mechanisms by IH [19]. From our study, IH exposure increases IL-8, ON (osteonectin), and MN mRNA levels in mammalian muscle cells and octamer binding transcription factor 1 (OCT1) is a key factor for the IH-induced upregulation of IL-8 and MN mRNA expression levels and that nuclear factor erythroid 2-related factor 2 (NRF2) serves as an essential factor for the IH-induced upregulation of ON mRNA expression [31] (Figure 3). No other study has examined the direct relationship between myokine and IH; therefore, further research is desirable.

Cardiomyokines are proteins secreted by a healthy or a diseased heart, and they perform a beneficial autocrine/paracrine function [98,99,100]. A search of PubMed (https://pubmed.ncbi.nlm.nih.gov/) (accessed on 1 November 2021) for “cardiomyokine” turned up only six articles. We exposed rat H9c2 cardiomyocytes and DMSO-induced cardiomyocytic differentiated mouse P19.CL6 cells to normoxia, IH, or SH for 24 h. We measured the mRNA levels of several kinds of cytokines and Reg family genes because Reg family genes were reportedly expressed under acute ischemic conditions in rat and human heart [101]. IH significantly increased the mRNA levels of Reg IV and hepatocyte growth factor (Hgf) in the cardiomyocytes, and the gene expression of Reg IV and Hgf was increased via downregulation of the miR-499 level in IH-treated cardiomyocytes. It is suggested that, in SAS patients, the upregulation of Reg IV and Hgf may function against the apoptosis of cardiomyocytes, leading to the maintenance of cardiac functions, and that miR-499 could play crucial roles in the regulation of these gene expressions [102].

## 3. Differences in Experimental IH Conditions In Vitro and In Vivo

Experimental models of IH, both in vitro and in vivo, include a variety of oxygen concentrations and exposure times. We and others have previously reported that the magnitude of IH expressed by SpO_2_ fluctuates between 75–98% and 50–80% in SAS [17,103,104], which is almost equivalent to the medium condition in our studies. Under our experimental ‘IH’ or ‘normoxia’ condition, cells were exposed either to IH (5 min [5% CO_2_ 1% O_2_]/10 min [5% CO_2_ 21% O_2_]) or normoxia for 24 h [5,7,19,25,32]. Several papers have been published that attempt to elucidate the IH mechanism using cell culture, but the IH exposure regimen has been arbitrary. The oxygen concentration of cells exposed to IH varies from 1% to 5%, and the exposure time varies from 30 s to 5 min; the oxygen concentration of normoxia is generally 16–21%, and the exposure time is 5–10 min. The conditions are set to repeat IH and normoxia for 4 to 48 h [77,105,106,107]. We recognize that the cell culture model of IH is different from the pattern of IH seen in OSAS patients in terms of the duration and frequency of episodes. Furthermore, it is very difficult to accurately reproduce IH in OSAS patients, and specifically, the level of intracellular oxygenation during apnea is unknown [77]. On the other hand, in vivo models of IH are being established that more closely resemble actual SAS conditions in humans. In in vivo, using mainly rats and mice, the oxygen concentration under IH conditions is often 5–12.5%, while that under normoxia is 21%, and IH and normoxia are repeated for 30 s each. Alternatively, IH is employed for 20 s, followed by a 5% oxygen concentration for 15 s and then rapid reoxygenation to room air levels. In most cases, IH is performed for 8–12 h per day and normoxia for 12–16 h per day, and the total number of days varies slightly from 6 to 24 weeks depending on the experiment [69,108,109]. Thus, in vivo experiments were conducted under conditions more similar to SAS.

The actual changes in oxygen levels in peripheral organs when animals are exposed to IH conditions are shown; hypoxia-reoxygenation occurs in response to IH exposure in the liver, adipose tissue, and skeletal muscle, which we review here [12,110]. Reinke et al. revealed the pattern of oxygen variability in various tissues during intermittent hypoxia. They found that hypoxia-reoxygenation occurs in the liver and skeletal muscle, while white adipose tissue shows slightly less amplitude and continuous hypoxia (thought to be due to relatively poor perfusion of adipocytes). They clarified the oxygenation of peripheral organs and established an in vivo IH model [12].

## 4. Intermittent Hypoxia and Inflammatory Cytokines

**Figure 4:**
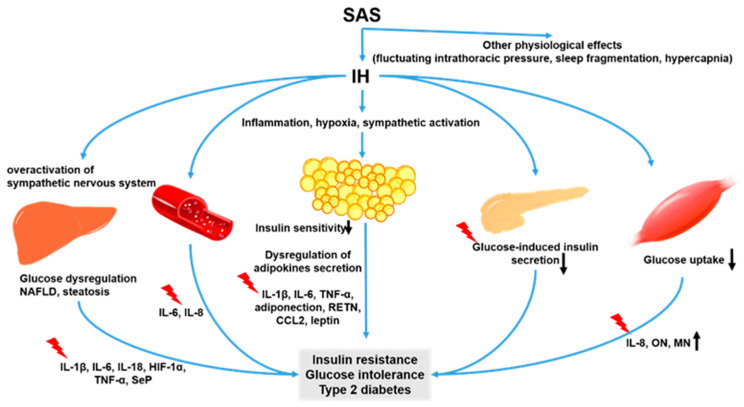
IH-induced dysfunctions in adipose tissues, skeletal muscle cells, and hepatocytes. Adipose tissues, skeletal muscle, and the liver are the main endocrine organs producing adipokines, myokines, and hepatokines, respectively. IH stimuli induce the expression/secretion of cytokines, which induce and/or worsen insulin resistance, glucose intolerance, and T2DM in adipocytes, skeletal muscle cells, and hepatocytes. IH also inhibits glucose-induced insulin secretion via the downregulation of CD38 [22] and vascular smooth muscle proliferation via IL-6-induced epiregulin expression [23,114]. IH promoted the production of TNF-α, IL-1β, and IL-6 and caused inflammation and cell apoptosis in pancreatic tissue via the MAPK signaling pathway [109]. Black up and down arrows mean increment and decrease, respectively.

In this review, we focus on the changes in cytokine expression under IH exposure, which suggest that inflammatory cytokines such as TNF-α and interleukins may be involved. It is thought that inflammation mediated by adipose tissue is particularly important in increasing insulin resistance in SAS patients [70,111,112]. However, inflammation in the liver, vascular endothelium, pancreas, and skeletal muscle was also implicated, suggesting that inflammation in these organs via cytokine secretion may exacerbate insulin resistance (Figure 4). The interactive effects of inflammatory cytokines secreted by each organ remain unclear, and further research is needed to clarify the mechanism of systemic inflammation. Furthermore, epigenetic mechanisms have been reported to induce inflammation under IH, including our results [5,7]. MiRNAs with altered expression levels in SAS patients have been reviewed by Chen et al., and their target genes are transcription factors, protein kinases, and proinflammatory cytokines [27]. However, the detailed mechanism of miRNA involvement in inflammation caused by IH exposure remains unclear [35], and further research is desirable.

## 5. Conclusions

In this review, we discussed the pathogenesis of IH, mainly in adipose tissue, the liver, and skeletal muscle, which leads to abnormal cytokine secretion and is associated with glucose intolerance (Figure 4). Abnormalities in cytokine secretion from the pancreas and vascular endothelium under IH conditions have also been studied [105,109,113], suggesting that IH exacerbates insulin resistance through a variety of complex mechanisms in various organs of the body.

## Abbreviations

ADIPOQ	Adiponectin
ANGPTL	Angiopoietin-like protein
AHSG	Alpha-2-HS-glycoprotein
CCL2	C-C motif chemokine ligand 2
C/EBP	CCAAT/enhancer binding protein
CHOP	C/EBP homologous protein
DM	Diabetes mellitus
FFA	Free fatty acid
FGF21	Fibroblast growth factor
HGF	Hepatocyte growth factor
HIF	Hypoxia-inducible factor
HIP/PAP	Hepatocarcinoma-intestine-pancreas/pancreatitis-associated protein
IH	Intermittent hypoxia
IL	Interleukin
I/R	Ischemia-reperfusion
LECT	Leukocyte cell-derived chemotaxin
LEP	Leptin
MAPK	Mitogen-activated protein kinase
miRNA	micro RNA
MN	Myonectin
NAFLD	Nonalcoholic fatty liver disease
NASH	Nonalcoholic steatohepatitis
NF-κB	Nuclear factor-κB
OCT1	Octamer DNA binding transcription factor-1
ON	Osteonectin
OSAS	Obstructive sleep apnea syndrome
PAI-1	Plasminogen activator inhibitor-1
PPAR	Peroxisome proliferator-activated receptor
Reg	Regenerating gene
ROS	Reactive oxygen species
SAS	Sleep apnea syndrome
SeP	Selenoprotein P
SH	Sustained hypoxia
TNF-α	Tumor necrosis factor-α
UCP-1	Uncoupling protein-1
VEGF	Vascular endothelial growth factor
WD	Western diet
